# Development and validation of a prognostic nomogram for 3-year all-cause mortality risk among elderly patients undergoing surgery for osteoporotic fractures

**DOI:** 10.3389/fmed.2024.1284207

**Published:** 2024-03-14

**Authors:** Chong Li, Qin Shi, Ya-qin Gong, Ting Zhang, Ke Lu

**Affiliations:** ^1^Department of Orthopedics, Affiliated Kunshan Hospital of Jiangsu University, Suzhou, Jiangsu, China; ^2^Department of Orthopedics, The First Affiliated Hospital of Soochow University, Orthopedic Institute of Soochow University, Suzhou, Jiangsu, China; ^3^Information Department, Affiliated Kunshan Hospital of Jiangsu University, Suzhou, Jiangsu, China; ^4^Chronic Disease Department, Kunshan Center for Disease Control and Prevention, Suzhou, Jiangsu, China

**Keywords:** prognostic model, mortality, nomogram, osteoporotic fractures, osteoporosis, 3 years

## Abstract

**Introduction:**

To develop and validate a comprehensive prognostic model for the mid-to-long term mortality risk among ≥50-year-old osteoporotic fracture (OPF) surgical patients.

**Methods:**

Our retrospective investigation included data from the Osteoporotic Fracture Registration System established by the Affiliated Kunshan Hospital of Jiangsu University, and involved 1,656 patients in the development set and 675 patients in the validation set. Subsequently, we employed a multivariable Cox regression model to establish a 3-year mortality predicting nomogram, and the model performance was further evaluated using C-index and calibration plots. Decision curve analysis (DCA) was employed to assess feasibility of the clinical application of this model.

**Results:**

Using six prognostic indexes, namely, patient age, gender, the American Society of Anesthesiologists (ASA) score, the Charlson comorbidity index (CCI), fracture site, and fracture liaison service (FLS), we generated a simple nomogram. The nomogram demonstrated satisfactory discrimination within the development (C-index = 0.8416) and validation (C-index = 0.8084) sets. Using calibration plots, we also revealed good calibration. The model successfully classified patients into different risk categories and the results were comparable in both the development and validation sets. Finally, a 1–70% probability threshold, according to DCA, suggested that the model has promise in clinical settings.

**Conclusion:**

Herein, we offer a robust tool to estimating the 3-year all-cause mortality risk among elderly OPF surgical patients. However, we recommend further assessments of the proposed model prior to widespread clinical implementation.

## 1 Introduction

Osteoporosis (OP) is a progressive skeletal disorder marked with reduced bone mass and widespread destruction of the bone tissue microarchitecture, which, in turn, enhances bone fragility and fracture susceptibility (1). An osteoporotic fracture (OPF) is a severe OP consequence that is mostly observed at the hip, vertebrae, distal forearm, and proximal humerus, but can also occur in the tibia, ribs, pelvis, clavicle, and fibula. Statistics indicate that OPFs are relatively common, with at least 1 fracture occurring during the lifetime of 40–50% females and 13–22% males (2). Alarmingly, within the Chinese population, over one-third women and around one-tenth men, aged ≥50 years of age, are likely to experience a major OPF in their remaining lifetimes (3). In particular, hip fractures are strongly correlated with enhanced mortality risk, with 33% males and 22% females with hip fracture expiring within the year (4, 5). The OPF-related economic burden is rather substantial on patients and the healthcare system. In fact, the yearly OPF incidence and expenditure are estimated to rise to 5.99 million fractures and $25.43 billion by 2050, respectively, in China alone (6). Hence, it is critical to restore patient ability to perform daily tasks at the pre-injury level in an attempt to reduce patient disability and mortality. Early surgical intervention is highly effective in achieving this goal (7–9), however patient physical reserve capacity and systemic complications must be thoroughly evaluated to obtain optimal therapeutic outcomes.

To ensure optimal patient prognosis, individual patients must be provided with personalized care. One critical aspect of managing OPF patients is the identification of high-risk patients who may be more prone to complications or death after surgery. Therefore, high-risk patient detection is critical to the provision of individualized care that may potentially enhance patient prognosis while minimizing physical and economic burdens of OPF patients. Prognostic models that incorporate multiple risk factors, such as, patient age, sex, comorbidities, and bone mineral density, were previously shown to assist clinicians in recognizing high-risk patients (10–15). Using these models, clinicals can accurately estimate postoperative patient mortality risk, and notify patients and their caregivers of the potential outcomes of surgery. Together, they can then formulate a personalized treatment plan that can best benefit the patient, based on the patient’s general condition and risk factors.

Although several OPF prognostic models have been proposed thus far, they have mostly involved prediction of hip fracture prognosis, and there are limited models that assess prognosis of other OPF sites. Furthermore, the available prognostic models have been criticized for their restrictive discriminative power (16), which may be due to the complexity of OPF pathophysiology as well as the multitude of associated risk factors. Additionally, certain variables employed in these models may be difficult to obtain, namely, patient fall history, bone quality, and frailty (17, 18). Moreover, a majority of existing models were developed based on a relatively small patient population (*n* < 1,000) (14, 15, 18), which may limit their generalizability to broader external clinical settings. In addition, there is a need for more accurate and reliable models that can predict mid-to-long term mortality in OPF patients. Despite the availability of prognostic models that evaluate in-hospital (14), 30-day (16), and 1-year mortality (12, 15), there is no current model for outcome prediction spanning >1 year.

To address the aforementioned limitations of the current OPF prognostic models, it is imperative to establish more comprehensive models that incorporate a wide range of comorbidities and risk factors to better predict mortality risk, particularly for outcomes beyond 1 year. Hence, herein, we established and validated an easy-to-use and widely applicable postoperative mid-to-long term mortality prognostic model for OPF surgical patients aged 50 years and older.

## 2 Methods

### 2.1 Data sources

In 2017, the Affiliated Kunshan Hospital of Jiangsu University (AKHJU) established the Osteoporotic Fracture Registration System (OPFRS) to improve care of OPF patients. OPFRS involves the prospective recording of patient profile, treatments administered, and prognosis. It is integrated with the Regional Health Registration Platform of the Kunshan City as well as the Population Death Registration System of the Jiangsu Province. This facilitates seamless data sharing across various healthcare systems.

The term “OPF” in the OPFRS specifically refers to fractures of the wrist, proximal humerus, hip, or vertebra that require hospitalization for surgical intervention. The diagnosis of OPF is based on the 10th revision of the International Statistical Classification of Diseases and Related Health Problems (ICD-10) terminology, which includes codes starting with S22, S32, S42, S52, or S72. OPF diagnosis was based on the 2017 Chinese recommendations of OPF diagnosis and treatment (19). OPF are low-energy or non-traumatic fractures that occur in daily life without significant external force or as a result of forces that would not typically cause a fracture. They are also referred to as fragility fractures. The term “forces that would not typically cause a fracture” refers to the forces exerted on the body from a standing height or lower, such as those resulting from a fall (19). It is important to note that the OPFRS excludes fractures caused by high-energy injuries. The system focuses specifically on capturing and analyzing data related to low-energy fractures associated with OP.

### 2.2 Patients

For this retrospective investigation, we analyzed patients aged 50 years or older who experienced a newly developed OPF. These patients required surgical treatment and were admitted to the hospital between January 2017 and July 2022. To ensure data completeness and accuracy, we excluded patients who died within 3 days of admission or prior to surgery, those with pathological fractures, and those with missing data. After applying these criteria, a total of 2,331 patients were recruited for our analyses.

**Figure 1:**
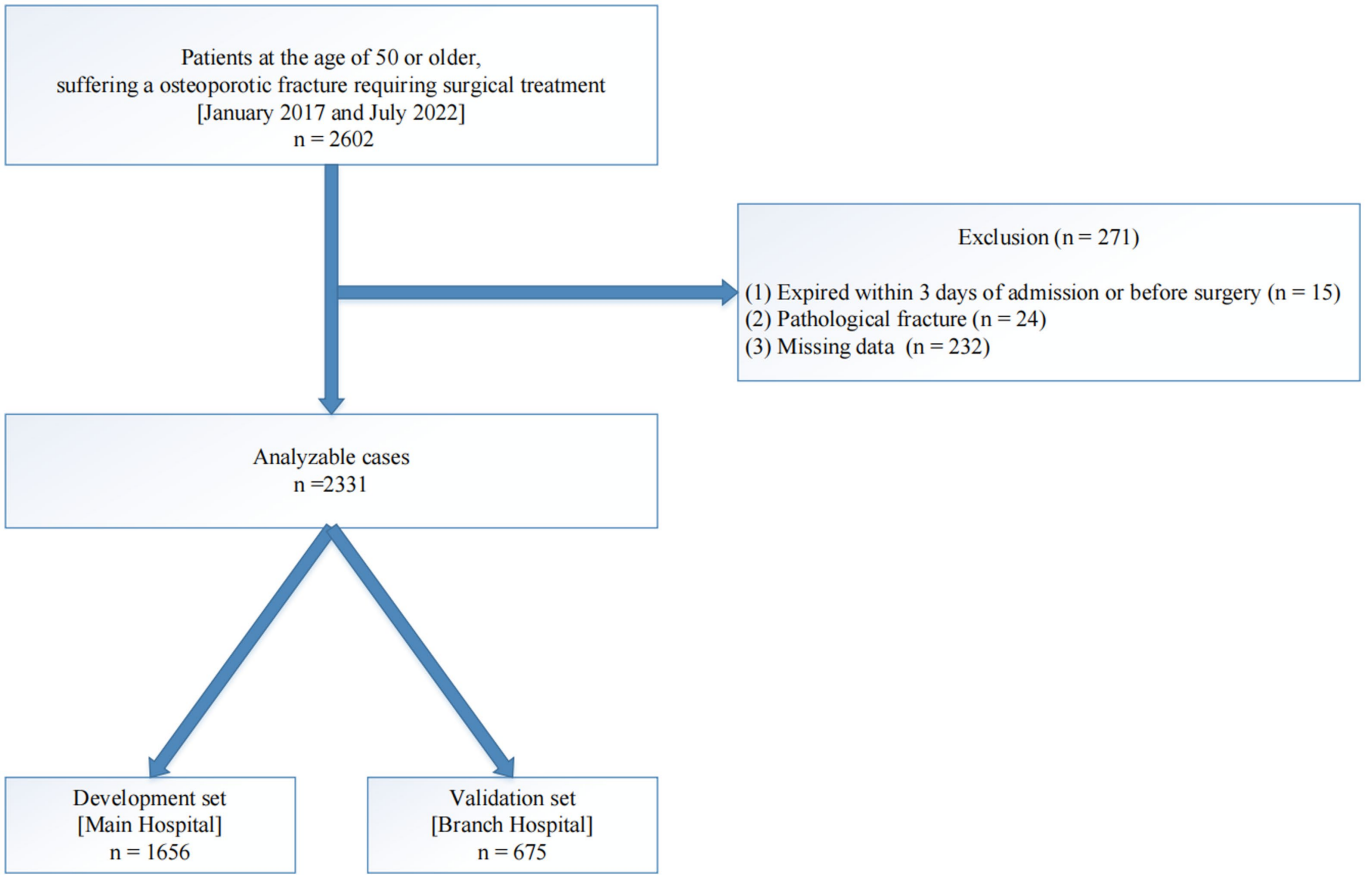
An illustration of the study flow chart.

Among the recruited patients, 1,656 were from the primary AKHJU hospital and were classified as the development set. Additionally, 675 patients from the You-yi branch of the AKHJU hospital were classified as the validation set (Figure 1). Ethical approval (approval number 2020-03-046-K01) was obtained from the participating institution, and written informed consent was obtained from all subjects prior to the study.

### 2.3 OPF management

All patients with OPFs were managed carefully in accordance with institutional guidelines, with direct guidance provided by physicians involved in their care. Patients underwent either a fracture liaison service (FLS) or routine osteoporosis management (No-FLS), as previously described in detail (20). The FLS program encompassed a comprehensive evaluation of bone health, high fracture risk patient identification, and initiation of appropriate treatment and follow-up. The No-FLS group were received standard OPF management, which primarily included pharmacological treatments and lifestyle modifications.

### 2.4 Dependent variable

The dependent variable was described as death due to any cause within a 3-year follow-up period starting from hospital admission. Data were censored after 3 years, at the time of moving out of the area, or at end of the investigation (September 3, 2022).

### 2.5 Prognostic indexes

Potential prognostic indexes included patient demographics, medication history, current medication usage, current diagnoses, comorbidities, surgical and other treatment data, anesthesia-related data, and laboratory data. Body mass index (BMI) represented the weight in kilograms divided by the square of the height in meters. The patient’s physical status was evaluated using the American Society of Anesthesiologists (ASA) score, which assesses the overall health status of the patient based on their physical fitness and presence of comorbidities (21). The ASA score ranges from 1 (healthy patient) to 5 (moribund patient who is not expected to survive without the surgery).

Comorbidity assessment was performed using the Charlson comorbidity index (CCI) (21). The CCI quantifies the burden of comorbid conditions by assigning a score to various medical conditions, with higher scores indicating a greater comorbidity burden. The CCI takes into account conditions such as myocardial infarction, congestive heart failure, peripheral vascular disease, cerebrovascular disease, dementia, chronic pulmonary disease, connective tissue disease, peptic ulcer disease, liver disease, diabetes, hemiplegia, renal disease, malignancy, and metastatic solid tumor.

Smoking status was described as a current or former smoker within the last 12 months, while drinking status was defined as drinking at least once a week for the prior 12 months. Prior fracture was described as any fracture prior to the index fracture date in the last 14 years (22). The fracture sites included fractures of the wrist, proximal humerus, femoral neck, femoral trochanteric or subtrochanteric, thoracic vertebrae, or lumbar vertebrae.

The laboratory data referred to the results of the patient’s examination upon admission, and included hemoglobin; circulating albumin, calcium, aspartate aminotransferase (AST), alanine transaminase (ALT), creatinine, urea nitrogen, and uric acid; as well as platelet, neutrophil, lymphocyte, and monocyte counts.

### 2.6 Statistical analysis

#### 2.6.1 Patients profiles

Patient demographics, as well as clinical, and laboratory profiles are listed with mean and standard deviation (SD) or median and quartile ranges (Q1–Q3) for continuous data, and frequency and percentage for categorical data. To evaluate differences in demographic profiles between the development and validation sets, we used Pearson’s chi-square or Fisher’s exact test for categorical variables and independent samples *t*-test and Mann–Whitney U test for normally and non-normally distributed continuous variables, respectively.

#### 2.6.2 Model development

Near-zero variance (NZV) analysis was employed to identify variables with little or no variability in their values. This was done to avoid overfitting while enhancing model performance. Following identification of the candidate prognostic indexes, multicollinearity among variables was examined using the variance inflation factor (VIF), with values ≥4 indicating potential collinearity. This step ensured that the model included only independent predictors. Finally, a multivariable Cox regression model was generated with the cph() function in R. The prognostic model was established using the backward stepwise variable selection based on the Akaike information criterion (AIC).

We generated two prognostic models for the development set using distinct sets of prognostic indexes: a clinical model that included only clinical prognostic indexes, and a laboratory model that additionally incorporated laboratory test results as prognostic indexes, thereby building upon the clinical model. We next compared the performances of both models via calculation of the integrated discrimination index (IDI) and net reclassification index (NRI). Subsequently, according to our findings, we established a simple nomogram, using the regression coefficients of the prognostic indexes (23, 24).

#### 2.6.3 Model validation

The nomogram performance was further validated using discrimination and calibration measurements within both development and validation sets. The model’s discrimination ability was assessed with the Harrell’s C-index (25), with values ranging between 0 and 1, where higher values indicate enhanced discrimination. Calibration was assessed via comparison of the estimated outcome probabilities with the actual probabilities using calibration plots.

Additionally, Kaplan–Meier survival curves were generated for individual risk groups based on the predicted probabilities from the Cox model (26). In this study, we obtained a practical risk spread by selecting four prognostic groups, using threshold at 16th, 50th, 84th percentiles of linear predictors. Lastly, we further assessed the hazard ratios (HRs) across risk groups, and log-rank tests were employed for the comparison of survival differences among various risk groups.

#### 2.6.4 Evaluation of clinical feasibility

We next determined the clinical feasibility of this prognostic model using DCA of the development set. DCA quantifies the net benefits of using the prognostic model at various threshold probabilities, which reflects the tradeoff between the potential benefits of intervening and the potential harms of unnecessary interventions (27). The net benefit was calculated by subtracting the proportion of false positives from the proportion of true positives, weighed by the relative harm of forgoing interventions compared with the negative consequences of an unnecessary intervention (28). In other words, DCA assesses the clinical utility of the prognostic model by comparing the benefits of using the model to guide clinical decision-making with the risks and costs of using the model in practice.

#### 2.6.5 Software

Empower Stats (www.empowerstats.com, X&Y solutions, Inc. Boston, MA) and R 4.2.0 (R Foundation for Statistical Computing, Vienna, Austria) were employed for all data analyses. A two-sided *p*-value <0.05 was set as the significance threshold.

## 3 Results

### 3.1 Patient profiles

In all, we analyzed 2,331 OPF patients, with an average age of 72.3 ± 10.6 years, and among which, 70.9% were females. During our 3-year follow-up period, 291 (12.5%) patients expired. The median follow-up duration was 27.0 months (Q1–Q3: 21.6–36.5 months). Table 1 summarizes the baseline clinical and laboratory profiles of the study participants. Based on our analysis, apart from CCI (*p*-value = 0.048), no obvious differences existed in other baseline profiles between the development and validation sets. This indicated that the study population was well-characterized and comparable between the development and validation sets, thus supporting the validity of the prognostic model that was established and verified in this study.

**Table 1:** Baseline clinical and laboratory characteristics of the study population by development and validation set (*N* = 2,331).

Characteristics	Development set	Validation set	Standardize diff.	*p*-value
	*n* = 1,656	*n* = 675		
	(*N*) Mean (SD) Median (Q1–Q3) / *N* (%)	(*N*) Mean (SD) Median (Q1–Q3) / *N* (%)		
Gender			0.02 (−0.07, 0.10)	0.74
Female	1,171 (70.71%)	482 (71.41%)		
Male	485 (29.29%)	193 (28.59%)		
Smoking			0.01 (−0.08, 0.10)	0.8
No	1,560 (94.20%)	634 (93.93%)		
Yes	96 (5.80%)	41 (6.07%)		
Drinking			0.02 (−0.07, 0.11)	0.59
No	1,609 (97.16%)	653 (96.74%)		
Yes	47 (2.84%)	22 (3.26%)		
Fracture site			0.11 (0.02, 0.20)	0.32
Thoracic vertebra	273 (16.49%)	93 (13.78%)		
Lumbar vertebra	452 (27.29%)	191 (28.30%)		
Wrist	73 (4.41%)	31 (4.59%)		
Proximal humerus	198 (11.96%)	67 (9.93%)		
Femoral neck	432 (26.09%)	188 (27.85%)		
Femoral trochanteric/subtrochanteric	228 (13.77%)	105 (15.56%)		
Surgical procedure			0.13 (0.04, 0.22)	0.69
Open reduction and internal fixation surgery for distal radius fracture	196 (11.84%)	65 (9.63%)		
Closed reduction and external fixation with a fixator for distal radius fracture	2 (0.12%)	2 (0.30%)		
Hemiarthroplasty of the hip joint	142 (8.57%)	69 (10.22%)		
Total hip arthroplasty	48 (2.90%)	14 (2.07%)		
Closed reduction and internal fixation surgery for proximal femur fracture	406 (24.52%)	176 (26.07%)		
Open reduction and internal fixation surgery for proximal femur fracture	59 (3.56%)	31 (4.59%)		
Percutaneous kyphoplasty	577 (34.84%)	225 (33.33%)		
Percutaneous vertebroplasty	79 (4.77%)	33 (4.89%)		
Closed reduction and internal fixation surgery for vertebral fracture	61 (3.68%)	25 (3.70%)		
Spinal fusion surgery	7 (0.42%)	2 (0.30%)		
Closed reduction and intramedullary nail fixation for proximal humerus fracture	7 (0.42%)	4 (0.59%)		
Open reduction and internal fixation for proximal humerus fracture	72 (4.35%)	29 (4.30%)		
Anesthesia			0.13 (0.04, 0.22)	0.06
General anesthesia	334 (20.17%)	154 (22.81%)		
Local anesthesia	478 (28.86%)	164 (24.30%)		
Spinal anesthesia	574 (34.66%)	240 (35.56%)		
Continuous epidural anesthesia	14 (0.85%)	12 (1.78%)		
Brachial plexus block anesthesia	256 (15.46%)	105 (15.56%)		
FLS			0.05 (−0.04, 0.14)	0.25
No	1,128 (68.12%)	443 (65.63%)		
Yes	528 (31.88%)	232 (34.37%)		
ASA score^a^			0.07 (−0.02, 0.16)	0.54
1	116 (7.00%)	42 (6.22%)		
2	1,070 (64.61%)	425 (62.96%)		
3	462 (27.90%)	206 (30.52%)		
4	8 (0.48%)	2 (0.30%)		
Prior fracture			0.07 (−0.02, 0.16)	0.1
No	1,354 (81.76%)	532 (78.81%)		
Yes	302 (18.24%)	143 (21.19%)		
Hypertension			0.03 (−0.06, 0.12)	0.51
No	1,361 (82.19%)	547 (81.04%)		
Yes	295 (17.81%)	128 (18.96%)		
Diabetes			0.01 (−0.08, 0.10)	0.79
No	1,577 (95.23%)	641 (94.96%)		
Yes	79 (4.77%)	34 (5.04%)		
CCI^b^			0.12 (0.03, 0.21)	0.048
0	1,469 (88.71%)	583 (86.37%)		
1	142 (8.57%)	67 (9.93%)		
2	36 (2.17%)	14 (2.07%)		
≥3	9 (0.54%)	11 (1.63%)		
Age, years	(1656) 72.03 (10.60) 72.00 (64.00–81.00)	(675) 72.82 (10.63) 73.00 (65.00–82.00)	0.08 (−0.01, 0.16)	0.1
BMI, kg/m^2^	(1656) 22.70 (3.36) 22.66 (20.36–24.84)	(675) 22.74 (3.32) 22.67 (20.53–24.82)	0.01 (−0.08, 0.10)	0.77
LOS, days	(1656) 10.04 (5.90) 9.00 (6.00–13.00)	(675) 9.98 (7.48) 9.00 (6.00–12.00)	0.01 (−0.08, 0.10)	0.83
Hemoglobin, g/L	(1633) 125.97 (18.22) 128.00 (116.00–139.00)	(665) 125.09 (18.81) 127.00 (114.00–138.00)	0.05 (−0.04, 0.14)	0.3
Serum albumin, g/L	(1620) 39.98 (4.22) 40.10 (37.40–42.70)	(664) 39.94 (4.37) 40.20 (37.50–42.70)	0.01 (−0.08, 0.10)	0.85
Serum calcium, mmol/L	(1641) 2.21 (0.13) 2.21 (2.13–2.29)	(669) 2.20 (0.14) 2.21 (2.13–2.29)	0.05 (−0.04, 0.14)	0.32
Platelet count, ×109/L	(1633) 176.42 (62.34) 170.00 (136.00–208.00)	(665) 176.51 (61.64) 166.00 (135.00–212.00)	0.00 (−0.09, 0.09)	0.97
Neutrophil count, ×109/L	(1633) 6.51 (3.04) 6.00 (4.34–8.10)	(665) 6.69 (3.37) 5.90 (4.40–8.10)	0.05 (−0.04, 0.14)	0.23
Lymphocyte count, ×109/L	(1633) 1.24 (0.54) 1.15 (0.87–1.50)	(665) 1.23 (0.52) 1.18 (0.90–1.50)	0.01 (−0.08, 0.10)	0.88
Monocyte count, ×109/L	(1633) 0.51 (0.27) 0.50 (0.30–0.62)	(665) 0.50 (0.23) 0.50 (0.35–0.60)	0.04 (−0.05, 0.13)	0.45
AST, U/L	(1642) 26.64 (27.21) 23.00 (18.00–28.00)	(669) 26.21 (14.21) 23.00 (18.00–29.00)	0.02 (−0.07, 0.11)	0.7
ALT, U/L	(1642) 23.90 (22.80) 19.00 (14.00–27.00)	(669) 22.61 (14.25) 19.00 (14.00–27.00)	0.07 (−0.02, 0.16)	0.18
Serum creatinine, μmol/L	(1642) 65.76 (26.86) 61.00 (52.00–73.00)	(670) 67.99 (48.24) 62.00 (51.00–74.00)	0.06 (−0.03, 0.15)	0.16
Serum urea nitrogen, mmol/L	(1642) 6.03 (2.47) 5.60 (4.55–6.90)	(669) 6.01 (2.54) 5.60 (4.50–6.89)	0.01 (−0.08, 0.10)	0.87
Serum uric acid, μmol/L	(1642) 284.14 (91.11) 272.00 (223.00–333.00)	(669) 284.51 (94.23) 273.00 (218.00–337.00)	0.00 (−0.09, 0.09)	0.93

a Higher scores indicate more severe physical status.

b Higher scores indicate more severe comorbidities.

SD, standard deviation; Q1, first quartile; Q3, third quartile; FLS, Fracture liaison service; ASA, American Society of Anesthesiologists; CCI, Charlson comorbidity index; BMI, body mass index; LOS, length of stay; AST, serum aspartate aminotransferase; ALT, serum alanine transaminase.

### 3.2 Establishment of model

Following NZV and multicollinearity assessment, the final clinical model included six indexes: age, gender, ASA score, CCI, fracture site, and FLS, whereas, the laboratory model included two extra prognostic indexes, AST and serum calcium, in addition to the clinical model (Table 2).

**Table 2:** Multivariable logistic regression models of 3-year mortality in the development set.

Predictors	Clinical Model		Laboratory Model	
	Coefficients	*p*-value	Coefficients	*p*-value
Age	0.08	<0.0001	0.08	<0.0001
ASA score				
1	Reference			
2	−0.55	0.30	−0.53	0.32
3	0.14	0.79	0.18	0.74
4	0.96	0.20	1.18	0.11
Gender				
Female	Reference			
Male	0.42	0.003	0.42	0.003
CCI				
0	Reference			
1	0.43	0.02	0.42	0.02
2	1.00	0.001	1.09	0.0002
≥3	1.60	<0.0001	1.46	<0.0001
Fracture site				
Thoracic vertebra	Reference			
Lumbar vertebra	0.43	0.19	0.32	0.33
Wrist	−0.44	0.56	−0.48	0.53
Proximal humerus	−0.10	0.85	−0.16	0.76
Femoral neck	0.98	0.001	0.94	0.001
Femoral trochanteric/subtrochanteric	0.69	0.03	0.63	0.04
FLS				
No-FLS	Reference			
FLS	−0.43	0.01	−0.42	0.01
Serum calcium, mmol/L	NA	NA	−0.81	0.12
AST, U/L	NA	NA	−0.01	0.07

FLS, fracture liaison service; ASA, American Society of Anesthesiologists; CCI, Charlson comorbidity index; AST, serum aspartate aminotransferase.

Table 3 compares the clinical and laboratory models of the development set. The aforementioned models were assessed based on their C-index, IDI, continuous-NRI, and median improvement in risk score. The C-index values of both models were 0.8416 (95% CI: 0.8181 to 0.8651) and 0.8430 (95% CI: 0.8200 to 0.8661), respectively. The laboratory model exhibited a small but statistically significant improvement in IDI, relative to the clinical model (0.0050 vs. reference, *p*-value = 0.07). However, we observed no substantial difference in the continuous-NRI between the analyzed models (0.0960 vs. reference, *p*-value = 0.10). The median improvement in risk score was statistically significant for the laboratory model, in relation to the clinical model (0.0050 vs. reference, *p*-value = 0.02). Taken together, the laboratory model exhibited some improvement in predictive performance, relative to the clinical model, although the differences were generally small, and some were not statistically significant.

**Table 3:** Comparisons of the clinical model and the laboratory model in the development set.

	Clinical model	Laboratory model	*p*-value
C-index (95% CI)	0.8416 (0.8181, 0.8651)	0.8430 (0.8200, 0.8661)	
IDI (95% CI)	Reference	0.0050 (−0.0001, 0.0170)	0.07
Continuous-NRI (95% CI)	Reference	0.0960 (−0.0190, 0.1880)	0.10
Median improvement in risk score	Reference	0.0050 (0.0001, 0.0150)	0.02

IDI, integrated discrimination index; NRI, net reclassification index; CI, confidence interval.

**Figure 2:**
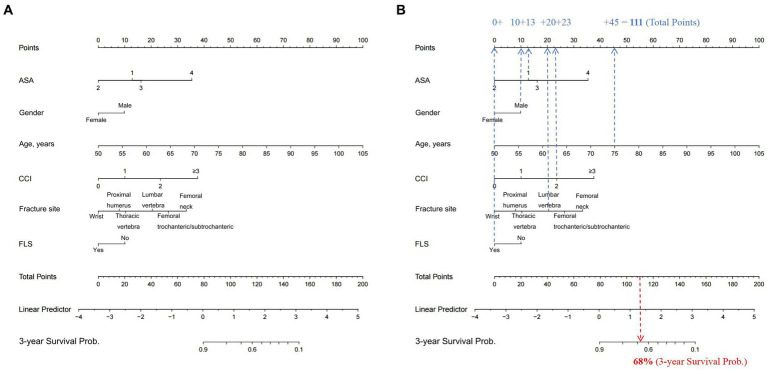
**(A)** Nomogram for the prediction of the 3-year OS of OPF patients, based on their corresponding preoperative clinical features. To use the nomogram, clinicians only need to identify the patient’s values on each variable axis, and then draw a vertical line between the points axis to obtain the corresponding predictor points for all variables. The total predictor points are the sum of all six indexes, which correspond to a specific estimated 3-year OS probability presented on the bottom axis of the nomogram. For illustrative purposes, consider the following scenario depicted in **(B)**: a male patient (awarded 10 points) is 75 years old (45 points), has recently sustained a lumbar vertebral OPF (20 points), is enrolled in a FLS (0 point), has a CCI score of 2 (23 points), and is classified as ASA score 1 (13 points). The aggregate of these prognostic indexes—10 + 45 + 20 + 0 + 23 + 13—amounts to a total score of 111. This cumulative score corresponds to a predicted 3-year mortality risk of 68% as indicated on the nomogram. OS, overall survival; OPF, osteoporotic fracture; FLS, Fracture liaison service; ASA, American Society of Anesthesiologists; CCI, Charlson comorbidity index.

Following evaluation of the clinical and laboratory models and their respective clinical application, we next established a simplified nomogram that balanced prognostic accuracy with the practicality of clinical usage. After extensive analysis, we based our nomogram solely on the clinical model, since it exhibited comparable predictive performance, relative to the laboratory model. The final nomogram (Figure 2A) included six simple prognostic indexes, namely, patient age, gender, ASA score, CCI, fracture site, and FLS, all of which are routinely measured in clinical setting, and can be easily obtained.

To use the nomogram, clinicians only need to identify the patient’s values on each variable axis, and then draw a vertical line between the points axis to obtain the corresponding predictor points for all variables. The total predictor points are the sum of all six indexes, which correspond to a specific estimated 3-year overall survival (OS) probability presented on the bottom axis of the nomogram.

For illustrative purposes, consider the following scenario depicted in Figure 2B: a male patient (awarded 10 points) is 75 years old (45 points), has recently sustained a lumbar vertebral osteoporotic fracture (OPF) (20 points), is enrolled in a FLS (0 point), has a CCI score of 2 (23 points), and is classified as ASA score 1 (13 points). The aggregate of these prognostic indexes—10 + 45 + 20 + 0 + 23 + 13—amounts to a total score of 111. This cumulative score corresponds to a predicted 3-year mortality risk of 68% as indicated on the nomogram (Figure 2B).

### 3.3 Model validation

**Figure 3:**
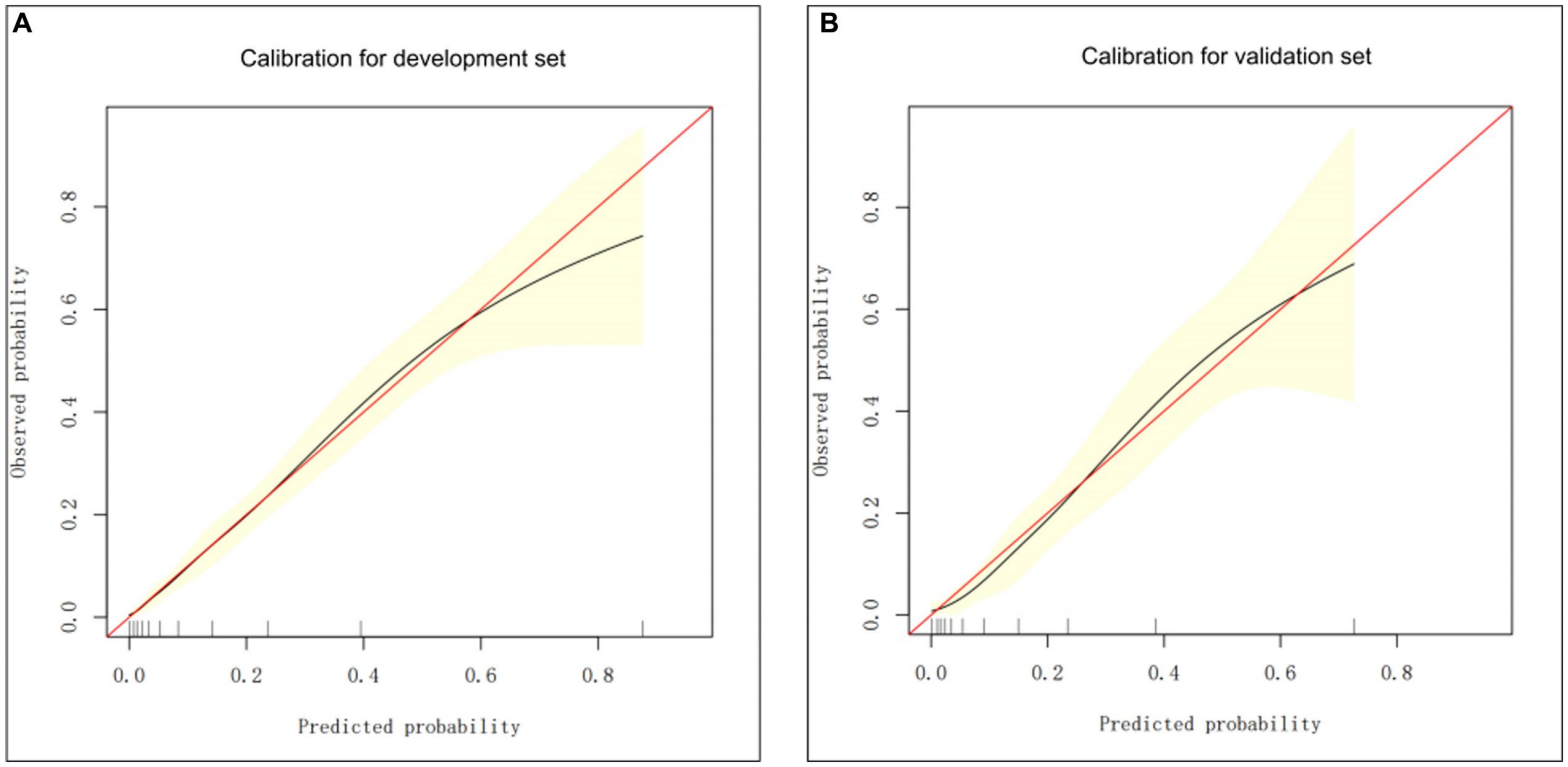
The calibration curve-based assessments of the clinical model calibration using the development **(A)** and validation **(B)** groups.

The nomogram performance was satisfactory in the validation set, as depicted by the Harrell’s C-index value of 0.8084, suggesting good discriminative ability. Additionally, the calibration plot presented in Figure 3 indicate that the predicted probabilities are in good agreement with the observed probabilities, thus providing evidence of good calibration.

**Figure 4:**
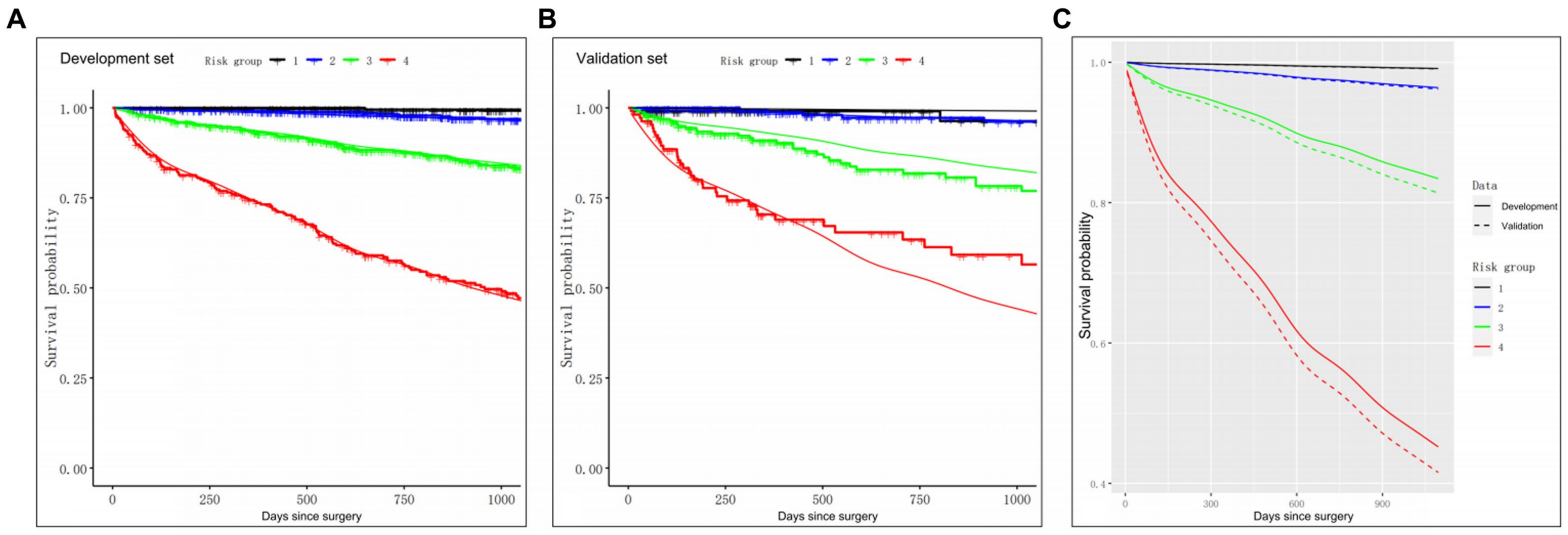
The Kaplan–Meier curves for the 3-year OS of patients in four distinct risk categories, based on the clinical model. **(A)** Calibration of OS probabilities in the development set. **(B)** Calibration of OS probabilities in the validation set. **(C)** The Kaplan–Meier curves of four risk groups in the development (solid lines) and validation (dash lines) groups. Smooth lines: 3-year OS as predicted in the development and validation sets from the prognostic indexes. Jagged lines: the Kaplan–Meier estimates involving the four risk groups. OS, overall survival.

Subsequently, we stratified the patients according to the Cox model-based probabilities into low, lower or higher intermediate and high-risk groups. In addition, using the aforementioned risk stratification, we generated the 3-year OS Kaplan–Meier curves for both the development and validation sets, as depicted in Figure 4. Plot inspection revealed several crucial insights. Firstly, both sets of four curves were well-separated, indicating good discrimination in both datasets. Secondly, the model displayed comparable discrimination in both the development and validation sets, however, the model was less effective in distinguishing between the low and lower intermediate groups in both sets. Thirdly, there was some deviation of the predicted OS curves from the observed curves after the 20-month follow-up phase in the validation set. This observation indicated that the model’s predictions may suffer from certain degree of miscalibration, which may be considered while interpreting the results.

To further evaluate the model’s performance, we further calculated the HRs between the four risk groups, as is presented in Table 4. The HRs observed in the development set were consistent with those in the validation set, indicating that the trends in risk ratios between the different groups were similar across both sets., thus confirming the reliability of the model’s predictions. Additionally, log-rank tests were performed to assess OS differences among the four risk groups. Our analysis revealed no obvious differences in OS between the low and lower intermediate groups (*p*-value = 0.08 for the development set, and *p*-value = 0.99 for the validation set). In contrast, we observed marked differences in OS among other three groups (all *p*-values <0.05), thereby suggesting that the prognostic model accurately stratified patients into distinct risk categories based on their preoperative clinical features.

**Table 4:** Discrimination measures and hazard ratios evaluated in the development and validation sets.

	HR	Low 95%CI	High 95%CI	*p*-value
Development set				
HR: group 2 versus 1	6.21	0.81	47.51	0.08
HR: group 3 versus 1	39.26	5.46	282.27	0.0003
HR: group 4 versus 1	167.92	23.47	1201.58	<0.0001
Validation set				
HR: group 2 versus 1	1.01	0.20	5.19	0.99
HR: group 3 versus 1	7.61	1.83	31.69	0.005
HR: group 4 versus 1	19.51	4.69	81.14	<0.0001

The risk groups were categorized into four groups based on the percentiles of the linear predictors: group 1 as the low-risk group, group 2 as the lower intermediate risk group, group 3 as the higher intermediate risk group, and group 4 as the high-risk group. The risk groups were divided at the 16th, 50th, and 84th percentiles of linear predictors. HR, hazard ratio; CI, confidence interval.

### 3.4 Evaluation of clinical feasibility

**Figure 5:**
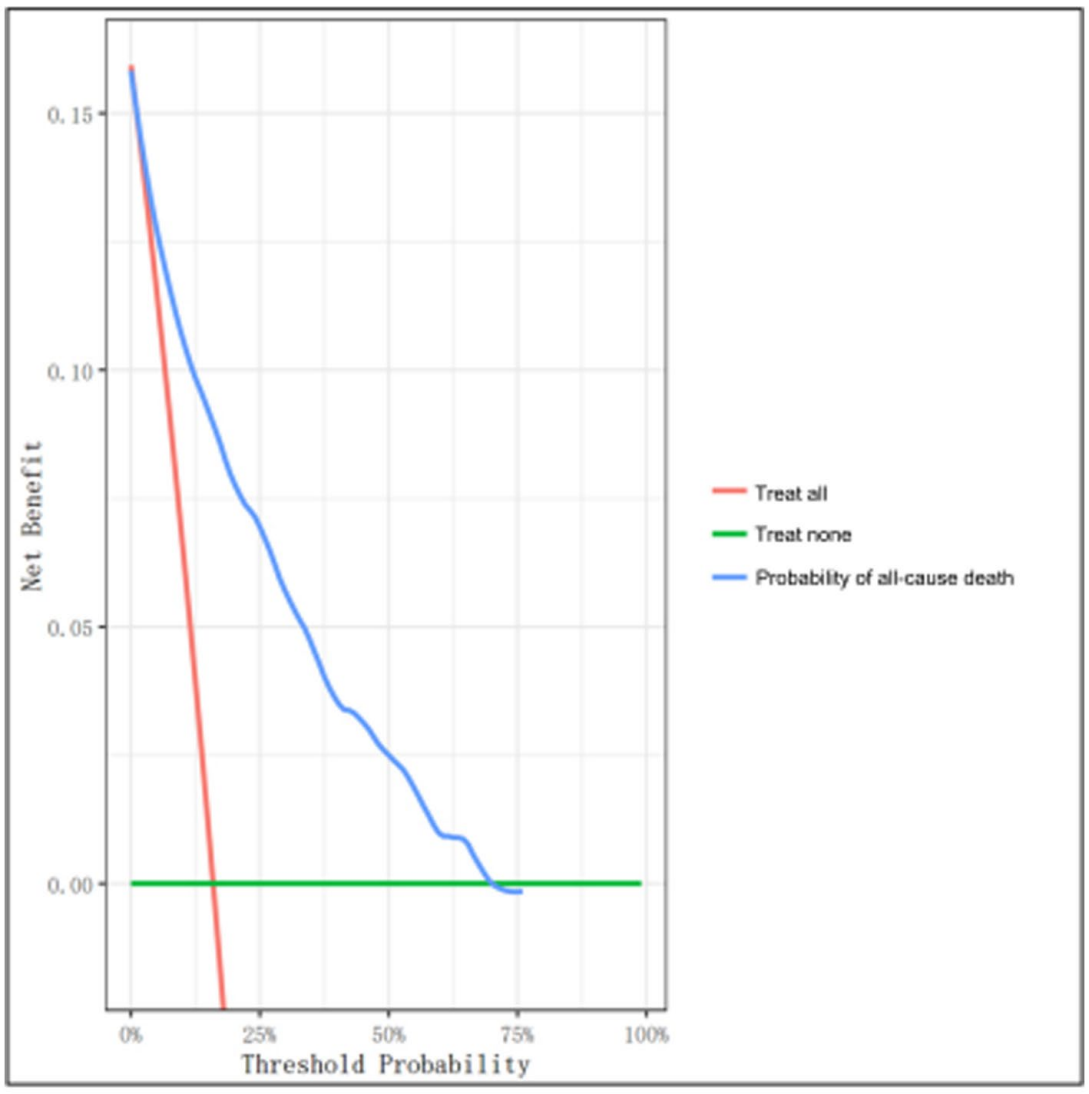
Decision curve analysis (DCA) of the clinical model using the development set. The y- and x-axes represent the net benefit and threshold probability, respectively. The threshold probability represents the probability at which the expected treatment benefit equals the expected benefit of avoiding treatment. The blue line denotes the nomogram. The results demonstrate that applying this nomogram can potentially yield net benefits at threshold probability ranges of 1–70%, as evidenced by the decision curve that lies above both the all-treatment (orange line) and the no-treatment (green line) curves.

DCA was employed for the assessment of clinical feasibility of the proposed model using the development set (Figure 5). The probability threshold for the 3-year all-cause mortality was plotted on the x-axis, while the standard net benefit of model usage was plotted on the y-axis. Based on our findings, the threshold probabilities 1–70% were considerably more beneficial than either the intervention-all or intervention-none approaches. These results suggest that the proposed model can effectively assist clinicians in making individualized treatment decisions based on the patient’s risk level. Specifically, when the predicted 3-year mortality risk is within the range of 1–70%, using the model to guide clinical decision-making can result in a higher net benefit than either treating all patients or treating none.

## 4 Discussion

Nomograms have become increasingly popular as prognostic tools in oncology and medicine owing to their user-friendly digital interfaces, augmented precision, and comprehensible prognoses, which can guide optimal treatment planning (29, 30). This study introduces a novel application of a nomogram designed to predict 3-year all-cause mortality in elderly patients undergoing surgery for OPF. Built with six readily available clinical variables, this nomogram offers a practical, evidence-based tool that clinicians can easily integrate into their workflow. The nomogram’s user-friendly format assists in calculating an individual patient’s risk, thereby informing and personalizing treatment plans. To illustrate its practical use, we provide an example where a clinician can quickly interpret the computed total score from the nomogram to stratify patients into different risk categories, guiding both immediate and long-term treatment decisions. Additionally, we employed external validation to confirm the model’s satisfactory discrimination and calibration power, which, in turn, validated its potential in clinical practice. Based on our OS analysis, there was marked differences in OS among the four risk groups. This highlights the strong and precise ability of the nomogram to classify patients according to their preoperative clinical characteristics. This information can be especially beneficial in guiding clinical decision-making, and in optimizing treatment regimen for patients at different risk statuses.

To optimize the nomogram’s utility for clinicians, we propose the following didactic application: Upon a patient’s admission, the clinician gathers the necessary variables—sex, age, OPF type, FLS involvement, CCI, and ASA score. These variables are marked on the nomogram, and a total predictor score is calculated. The clinician then interprets this score against the nomogram’s mortality risk scale. For instance, a higher total score would suggest a higher risk of mortality, prompting the clinician to consider more aggressive interventions or closer monitoring. This process is quick, straightforward, and can be seamlessly incorporated into clinical practice, making the nomogram a valuable tool for tailoring patient care strategies.

The proposed nomogram employs readily available indexes that are typically obtained upon admission or early treatment in the emergency department, namely, ASA score and CCI. The predictive performances of the ASA and CCI scores were earlier demonstrated in numerous studies, including a systematic review and meta-analysis by Smith et al. (31), as well as a meta-analysis by Hu et al. (32), both of which revealed that higher ASA and CCI scores were strong OS indicators at 12 months following hip fracture surgery (33, 34). These scores were also shown to estimate patient OS following adult spinal deformity surgery. Our analysis consistently demonstrated that higher CCI and ASA scores were intricately linked to enhanced risk of relatively long-term mortality. Although the CCI and ASA scores may be unmodifiable risk predictors, the aforementioned findings can aid in generating more precise prognosis prediction of mortality risk following OPF surgery.

Unlike the CCI and ASA scores, FLS was included in our prognostic model as a modifiable risk predictor. FLS is a coordinated care model that encompasses patient identification, education, risk assessment, intervention, and long-term monitoring (35), therefore, it is an essential component of our model. The FLS application is widely reported to strongly reduce mortality rates among elderly OPF patients (35–37), thereby highlighting the significance of this intervention in patient prognosis. FLS incorporation into our prognostic model facilitated a more comprehensive approach to managing OPF patients. FLS enables clinicians to recognize patients at high risk of experiencing future fractures and mortality, this is critical for administering appropriate and timely interventions, such as, pharmacological intervention and lifestyle modifications, that minimizes the possibility of future fractures or surgery-related death. Such personalized treatment can potentially optimize FLS effectiveness, and result in enhanced patient outcomes and reduced healthcare costs.

Multiple risk prediction tools are available for use in orthopedic surgery, and they have associated strengths and limitations. The O-POSSUM (38) and E-PASS (11) scores are widely established for risk stratification, however, their complex calculations and laboratory or intra-operative value requirements restrict their practicality in routine clinical practice. Likewise, the NHFS (39) and HULP-HF (18) scores are strong predictors of 30-day OS following hip fracture surgery, however, they require additional information that are often not readily available. In addition, AHFS (17) was earlier validated for detecting patients at a high mortality risk following hip fracture, however, it also requires the Parker Mobility Score (PMS) calculation and NHFS. Contrary to the aforementioned prediction tools, the present study employs a Cox model to establish a relatively long-term mortality predictive tool for OPF patients. This model provides valuable information regarding patient prognoses beyond the immediate post-operative or even one-year follow-up period. Additionally, this study utilizes readily available predictive factors commonly obtained in routine clinical settings, which encompass the key main categories of OPFs. Together, these factors strengthen the model’s practicality and feasibility in clinical setting.

The accurate stratification of OPF patients into distinct risk categories based on their preoperative clinical features is extremely beneficial particularly in clinical decision making and optimizing personalized treatment regimen for patients at varying levels of risk. Early detection of patient risk levels can effectively guide clinicians to the most appropriate treatments, follow-up care, and rehabilitation strategies for this population, which may ultimately enhance patient outcomes as well as overall quality of care provided in the context of OPFs. Additionally, employing the proposed nomogram in clinical practice can potentially facilitate efficient allocation of healthcare resources while identifying high-risk patients who may benefit from more aggressive treatment and closer monitoring. Consequently, this could lead to augmented patient care, reduced complications, and lower healthcare costs related to OPF treatment.

Our work has certain strengths that contribute to its value in the field of OPF management. Firstly, we introduced a relatively long follow-up duration, a median of 27.0 months, which allowed for a more accurate assessment of patients’ outcomes and OS probabilities. Secondly, we included a diverse range of OPFs, encompassing five distinct categories of fractures, which enhanced the generalizability of our findings. Thirdly, this study involved a large sample population of 2,331 patients, which provided a robust basis for the establishment and verification of the proposed prognostic nomogram. Fourthly, the rigorous statistical analysis and extensive assessment of the model’s performance and clinical feasibility added to the study’s strengths, and simultaneously enhanced the reliability of the nomogram-based predictions. Lastly, during the prognostic nomogram establishment, we considered both clinical and laboratory indexes, and a comparison was made between the two models.

However, this study also has a limitation. Firstly, our sample population only included surgical patients, so our nomogram may have limited predictive power towards elderly OPF patient who do not undergo surgery. Secondly, this was a single center study, which may restrict its generalizability to other populations and settings. Thirdly, certain relevant factors that may influence patients’ outcomes, such as, patient psychology and social health determinants, were not analyzed in this study. Hence, we warrant additional investigations that include these factors to provide a more comprehensive assessment of the patient risk profiles. Finally, our assessment of clinical feasibility was solely based on DCA, which may not fully encapsulate the practical considerations and costs associated with model implementation in clinical settings. Additional investigations are necessary for a more thorough examination of our model, particularly, in terms of its clinical feasibility, such as, cost-effectiveness analysis, as well as its impact on clinical decision-making and patient outcomes.

## 5 Conclusion

Herein, we propose a valuable tool for predicting the 3-year all-cause mortality of elderly OPF surgical patients. Our prognostic nomogram has an easy-to-use format and relies on readily available clinical variables, which make it a promising tool for clinical decision-making and patient counseling. However, we recommend further assessments of the proposed model prior to widespread clinical implementation.

## Data availability statement

The raw data supporting the conclusions of this article will be made available by the authors, without undue reservation.

## Ethics statement

The studies involving humans were approved by Ethics Committee of Kunshan Hospital, Affiliated with Jiangsu University. The studies were conducted in accordance with the local legislation and institutional requirements. The participants provided their written informed consent to participate in this study.

## Author contributions

CL: Writing – original draft, Methodology, Investigation, Funding acquisition, Formal analysis, Conceptualization. QS: Writing – review & editing, Validation, Supervision, Methodology, Conceptualization. Y-qG: Writing – review & editing, Validation, Software, Project administration, Methodology, Formal analysis, Data curation. TZ: Writing – review & editing, Data curation. KL: Writing – review & editing, Writing – original draft, Visualization, Project administration, Funding acquisition, Data curation, Conceptualization.
